# Is There Value in Performing Yearly Screening for Latent Tuberculosis Infection by Interferon-Gamma Release Assay Among Patients Living With HIV in Non-Endemic Settings?

**DOI:** 10.1093/ofid/ofag227

**Published:** 2026-06-19

**Authors:** Carlo Foppiano Palacios, Amit Achhra, Lydia Barakat, Michael Virata, Ritche Hao, John Baxter, Maricar Malinis

**Affiliations:** Division of Infectious Diseases, Department of Medicine, Cooper Medical School of Rowan University, Camden, New Jersey, USA; Section of Infectious Diseases, Department of Medicine, Yale School of Medicine, New Haven, Connecticut, USA; Section of Infectious Diseases, Department of Medicine, Yale School of Medicine, New Haven, Connecticut, USA; Section of Infectious Diseases, Department of Medicine, Yale School of Medicine, New Haven, Connecticut, USA; Section of Infectious Diseases, Department of Medicine, Yale School of Medicine, New Haven, Connecticut, USA; Division of Infectious Diseases, Department of Medicine, Cooper Medical School of Rowan University, Camden, New Jersey, USA; Division of Infectious Diseases, Department of Medicine, Vanderbilt University Medical Center, Nashville, Tennessee, USA

**Keywords:** HIV screening, latent tuberculosis, tuberculosis

## Abstract

We evaluated the effectiveness of annual LTBI screening via IGRA in people with HIV in non-endemic settings over 5 years. Out of 1898 patients with at least 2 IGRAs, only 12 developed new LTBI, highlighting the low yield of universal screening. Findings support targeted screening per current guidelines.

## Teaser

In a multicenter retrospective cohort of people living with HIV in a low tuberculosis incidence setting, routine annual IGRA screening yielded few true incident cases of latent tuberculosis infection and frequent false-positive conversions. These findings support current guideline-recommended targeted, rather than universal, annual LTBI screening. R&P Agreement (Rowan University)

## BACKGROUND

Worldwide, tuberculosis (TB) remains the leading cause of death among people with HIV-1 (PWH) [1]. In the U.S., TB incidence among PWH has declined and is the lowest ever reported [2]. Latent tuberculosis infection (LTBI) occurs when patients are exposed to *M. tuberculosis* but do not eliminate the viable bacilli, remaining quiescent for years [3]. Consistent with U.S. guidelines, we use the term LTBI. Notably, the World Health Organization now favors the terminology “tuberculosis infection” and “tuberculosis disease” over the traditional designations of “latent” and “active” [4, 5].

PWH have an increased risk of TB reactivation from LTBI, with an annual risk of 3–16% per year [6, 7]. While TB infection can occur at any CD4 T lymphocyte (CD4) cell count, the highest risk is in patients with lower CD4 counts and not on anti-retroviral therapy (ART) [8, 9]. Screening for LTBI is a component of routine care of PWH to identify at-risk patients and mitigate the risk of TB reactivation [10–12].

Current guidelines recommend that all PWH be initially screened for LTBI with either interferon-gamma release assays (IGRA) or tuberculin skin testing (TST) [13]. Repeat screening is indicated in either PWH with any new TB exposure risk (such as living in congregate setting, housing difficulties, travel to high-TB incidence country, incarceration, etc.) or an initial CD4 count <200 cells/mm^3^ after the initial negative screen when they have started ART and obtain a CD4 count ≥200 cells/mm^3^ [6]. Despite this, some centers continue to conduct annual IGRA testing in all PWH irrespective of new exposure risk [14]. However, the clinical utility of routine repeat LTBI screening with IGRA in PWH without new TB exposure risk, particularly in low-incidence settings, remains unclear, and practice patterns vary across centers. To address this gap, we evaluated the yield and utility of universal yearly LTBI screening by IGRA among PWH in clinics at 2 academic urban centers in non-endemic TB regions.

## METHODS

### Data Collection

We conducted an observational, retrospective study of PWH Yale New Haven Health and Cooper University Health Care from 2017 to 2021 by electronic medical record (EMR) review. We included patients who were ≥18 years of age at their initial clinic encounter and had undergone at least 2 IGRA assays done during study period. New LTBI diagnoses were determined through assessments by HIV providers. In clinical practice, LTBI diagnoses were typically established when a newly positive IGRA result was observed, especially if corroborated by subsequent positive IGRA testing or a history of lifetime epidemiologic exposure to tuberculosis without subsequent negative IGRA testing. LTBI diagnosis was made in the absence of clinical or radiographic evidence of active tuberculosis. IGRA results were interpreted using established clinical laboratory criteria in accordance with manufacturer recommendations for QuantiFERON-TB Gold Plus assays. Results were classified as positive, negative, or indeterminate according to the laboratory's reported interpretation. Over the course of the study, both sites transitioned from QuantiFERON-TB Gold to QuantiFERON-TB Gold Plus assays: Yale on 17 July 2018, and Cooper on 3 December 2018. Data on demographics, clinical variables including clinical LTBI diagnosis and TB risk factors based on their problem list in EMR, and annual IGRA results were collected. For patients who demonstrated IGRA seroconversion, additional detailed chart review was performed to identify further TB risk factors not consistently captured in structured EMR fields. TST results were not included in the analysis because TST testing is not universally documented in the EMR, resulting in incomplete capture for retrospective review. We utilized the Charlson Comorbidity Index as a standardized measure to quantify comorbidity burden for the purpose of baseline risk adjustment. Yale and Cooper Institutional Review Boards approved the study. De-identified data are available from the corresponding author upon reasonable request.

### IGRA Seroconversion Stratification

Patients were stratified into 3 groups based on IGRA seroconversion: those with negative to positive (Group A), indeterminate to positive (Group B), and negative to indeterminate (Group C). For patients with IGRA seroconversion, we also gathered data on age at seroconversion, Charleston comorbidity index (CI), additional risk factors for TB, chest imaging results, HIV clinical data, and treatment.

### Outcomes

The primary outcome was the proportion of patients converting from a negative to positive IGRA. Secondary outcomes included risk factors for LTBI diagnosis, proportion of patients with negative to indeterminate conversion of IGRA, and comparison of patients with negative to positive IGRA conversion versus patients with negative to indeterminate conversion.

### Statistical Analyses

Descriptive statistics, Fisher's Exact test, student's t-test, and Kruskal Wallis tests were performed. Missing data were addressed through available-case analysis, whereby each analysis was restricted to patients with complete data for the relevant variables. Exact 95% confidence intervals for proportions were estimated using the Clopper–Pearson method due to the small sample sizes in several comparisons. For patients with QuantiFERON-TB Gold Plus testing, the mean of their 2 TB mitogen values was calculated. LTBI incidence per 1000-patient years was calculated by multiplying the cases of new LTBI by 1000 and dividing by the product of 5 years (study time-frame) and total patients who underwent repeat IGRA testing. Given that only 12 patients met criteria for a new diagnosis of LTBI, we conducted a post-hoc power analysis to compare those with and without new LTBI diagnoses in the negative-to-positive IGRA seroconversion group (Group A). The estimated post-hoc power was approximately 61% for repeat positive IGRA results, 68% for history of travel to a TB-endemic area, and 91% for prior LTBI treatment, based on the observed sample sizes and effect sizes. We analyzed data using R version 4.0.2.

## RESULTS

### Patient Demographics and TB History

2694 PWH were in care but only 1898 patients underwent IGRA testing at least twice during the study period. Of the 1898, 1270 (66.9%) had ≥3 tests, 606 (31.9%) had ≥4 tests, and 119 (6.3%) had yearly testing. Among the 1898 included patients, 1249 (65.8%) were males, 962 (50.7%) were Black, and 447 (23.6%) were LatinX with 237 (12.5%) speaking a language other than English (Supplementary Table 1). Only 112 (5.9%) had a positive IGRA result, with the following annual rates: 32 (2.8%) in 2017, 25 (2.0%) in 2018, 32 (2.5%) in 2019, 35 (3.3%) in 2020, and 37 (3.6%) in 2021 (Supplementary Table 2).

### IGRA Seroconversion

Seventy-two patients (3.8%) had IGRA seroconversion: 39 in Group A, 1 in Group B, and 32 in Group C (Supplementary Table 3). Compared with Group A, Group C was more likely to have CD4 count <200 cells/mm^3^ before IGRA seroconversion (5.1% vs 34.4%, *P* = .002), higher mean HIV viral loads before IGRA seroconversion (2336 vs 101 893 copies/mL, *P* = .02), less likely to be on an INSTI-based regimen (71.8% vs 46.9%, *P* = .05), and more likely to be on an NNRTI-based regimen (2.6% vs 25.0%, *P* = .009) before seroconversion (Supplementary Table 4).

### Group A: Negative to Positive IGRA Seroconversions

Out the 39 patients, 12 patients (0.6%) with IGRA seroconversions were adjudicated as new LTBI. The remaining 27 were not diagnosed with new LTBI because their repeat IGRA tests were negative (59.3%, N = 16/27), lacked TB risk factors (55.6%, N = 15/27), and had a history of previously treated LTBI (29.6%, N = 8/27).

Compared with those without new LTBI diagnosis, patients with new LTBI were more likely to report travel to TB endemic areas (33.3% vs 3.7%, *P* = .03) and have a repeat positive IGRA (50.0% vs 14.8%, *P* = .04), and less likely to have previously been treated for LTBI (0% vs 29.6%, *P* = .04). We found no significant difference between patients with and without new LTBI diagnoses and the value of positive QuantiFERON (*P* = .37) or use of QuantiFERON-TB Gold Plus (*P* = 1.0, Table 1).

**Table 1.** Comparing Patients With New Positive IGRA With and Without New LTBI Diagnosis

	No New LTBI (N = 27) N (%, 95% CI)	New LTBI (N = 12) N (%, 95% CI)	*P V*alue
TB risk and predisposing factors			
Born in endemic country	N = 2/6 (33.3, 4.3–77.8)	N = 4/6 (66.7, 22.2–95.7)	.57
Travel to endemic country	1 (3.7, 0–19.0)	4 (33.3, 10.0–65.1)	.03
Incarceration	6 (22.2, 8.6–42.3)	3 (25.0, 5.5–57.2)	1.00
Homelessness	6 (22.2, 8.6–42.3)	2 (16.7, 2.1–48.4)	1.00
TB exposure	1 (3.7, 0–19.0)	0 (0, 0–26.5)	1.00
Smoking use	18 (66.7, 46.0–83.5)	7 (58.3, 27.7–84.8)	.72
Heavy alcohol use	7 (25.9, 11.1–46.3)	3 (25.0, 5.5–57.2)	1.00
Diabetes	7 (25.9, 11.1–46.3)	1 (8.3, 0.2–38.5)	.40
Malnutrition	3 (11.1, 2.4–29.2)	1 (8.3, 0.2–38.5)	1.00
Military service	1 (3.7, 0–19.0)	1 (8.3, 0.2–38.5)	1.00
Healthcare worker	1 (3.7, 0–19.0)	0 (0, 0–26.5)	1.00
Substance use history	8 (29.6, 13.8–50.2)	4 (33.3, 10.0–65.1)	1.00
Any TB risk factor	12 (44.4, 25.5–64.7)	8 (66.7, 34.9–90.1)	.35
IGRA			
QuantiFERON TB mitogen value (mean ± SD)	1.07 ± 1.44	0.70 ± 0.82	.37
QuantiFERON-TB Gold Plus performed	23 (85.2, 66.3–95.8)	11 (91.7, 61.5–99.8)	1.00
TB skin testing			
Done	14 (51.9, 31.9–71.3)	9 (75.0, 42.8–94.5)	.29
Positive	N = 8/14 (57.1, 28.9–82.3)	N = 3/9 (33.3, 7.5–70.1)	.66
Repeat IGRA			
Negative	16 (59.3, 38.8–77.6)	1 (8.3, 0.2–38.5)	.004
Positive	4 (14.8, 4.2–33.7)	6 (50.0, 21.1–78.9)	.04
Not done	7 (25.9, 11.1–46.3)	5 (41.7, 15.2–72.3)	.46
Past treated LTBI	8 (29.6, 13.8–50.2)	0 (0, 0–26.5)	.04

### New Latent TB Infections

**Figure 1.**
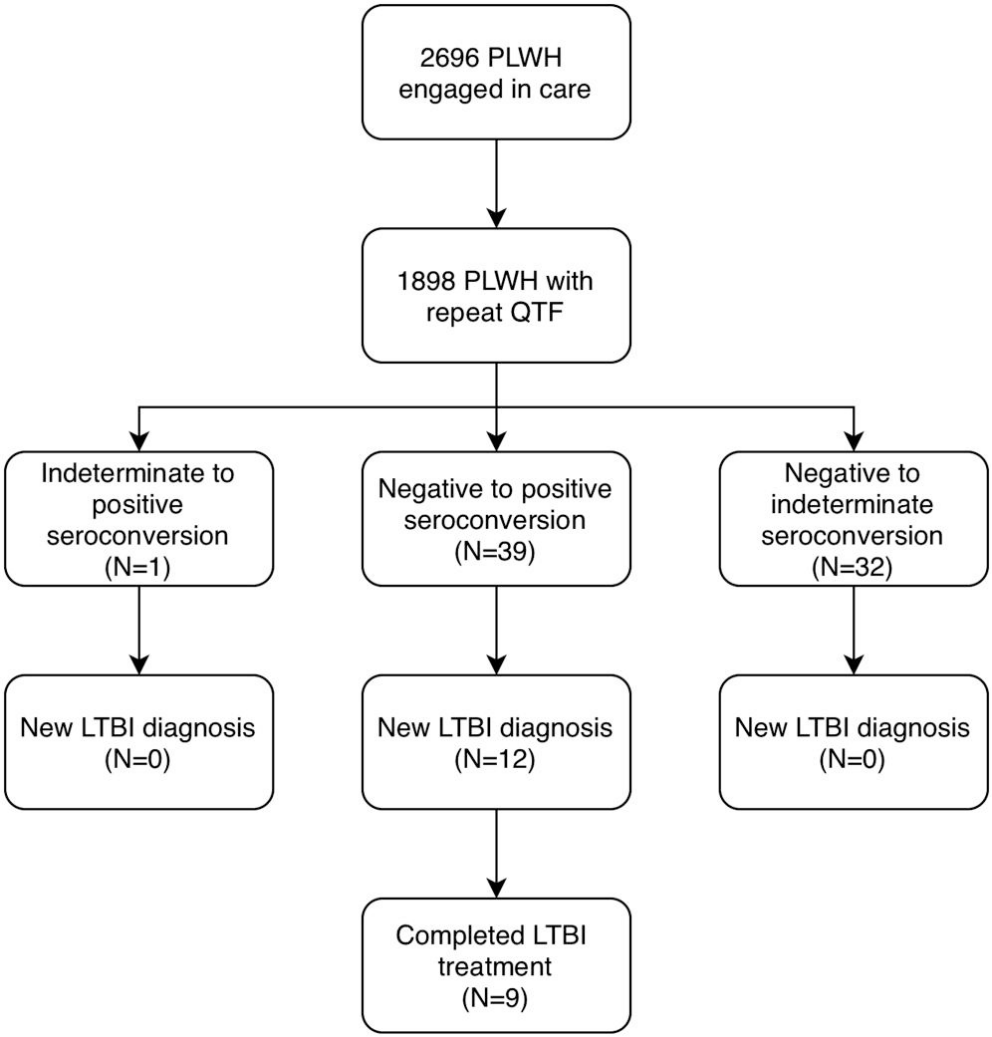
Flowchart of seroconversions, diagnosis, and treatment of LTBI.

Incidence of new LTBI was 1.3 cases/1000 patient-years. All patients with new LTBI had negative baseline IGRA (Group A). Of these, 8 PWH (67%) had ≥1 TB risk factor and none had CD4<200 cells/mm^3^ before IGRA seroconversion. Eleven (91.7%) patients were recommended for LTBI treatment (1 lost to follow-up), 10 (83.3%) started treatment (1 died prior to initiation), 9 (75%) patients completed treatment, and none developed tuberculosis (Figure 1).

## DISCUSSION

Among people with HIV with yearly IGRA testing, we sought to assess the rate of IGRA seroconversion in a non-TB endemic setting. Over a 5-year period involving 1898 patients, we observed only 39 cases of IGRA seroconversion from negative to positive. Moreover, only a few of those who experienced positive seroconversion were diagnosed with new LTBI, and the remaining majority were either false-positives or had previous LTBI or TB treatment history. Overall, the incidence of developing LTBI in PWH was rare, at 1.3 cases/1000 patient-years.

Role of yearly LTBI screening among PWH in TB non-endemic settings is unclear. IGRA offers advantages over TST due to need for only 1 visit for a blood draw and false-positive TSTs among patients with previous Bacillus Calmette-Guérin (BCG) vaccination [15, 16]. While we aimed to evaluate yearly IGRA tests, in practice, few patients underwent yearly IGRA testing, suggesting logistic challenges in implementing this approach. Upon repeat IGRA screening, only a third of patients (N = 12) with negative to positive IGRA seroconversion were diagnosed with new LTBI. One prior study among PWH over 3 years from Vienna (TB-nonendemic setting) found that IGRA conversions occurred in 9% of patients 24 months after initial testing, particularly among patients from TB endemic countries or with history of injection drug use [17]. Another study from Rome observed IGRA conversions in 1.8% of PWH several years after their initial negative IGRA [18]. Various studies of serial IGRA testing among healthcare workers (HCW) found common conversions, raising concerns about false-positive results and potential over diagnosis of LTBI [19–21]. In our cohort, remaining IGRA seroconversions were adjudicated as false-positive due to lack of TB risk factors, repeat negative IGRA, and previously treated LTBI. A prior study of from the US found that IGRA reversions were common in 72% of patients with a positive IGRA [22]. While previously mentioned studies from Rome and Vienna found positive to negative IGRA reversions in 26–33% of PWH upon repeat IGRA testing [17, 18]. Annual IGRA testing is no longer recommended for other populations, including HCW, unless there is a new TB exposure [23]. Instead, current guidelines recommend annual testing of PWH with a prior negative LTBI diagnostic test and new epidemiologic risk factors for TB [6]. Routine annual IGRA screening provided limited clinical benefit among PWH in this low-TB-incidence setting: among nearly 1900 patients undergoing repeat testing over 5 years, only 12 were adjudicated as newly diagnosed LTBI. Many observed IGRA seroconversions were attributable to false-positive results, subsequent negative tests, or past treated LTBI. Serial testing can generate additional healthcare costs through repeated laboratory testing, follow-up imaging studies, and clinical evaluations, as well as the management of cases prompted by false-positive results. These findings reinforce current recommendations advocating targeted, rather than universal, annual targeted LTBI screening.

Our study had several limitations. First, it was a retrospective review of EMR, limited by the available data, which may have led to incomplete identification of TB risk factors. Second, as the clinics had been following patients for years before EMR implementation, we could not document initial IGRA or TST results. Third, CD4 counts were abstracted from clinical records; however, these measurements were not consistently obtained at the time of IGRA testing. As a result, we were unable to systematically assess adherence to guideline-recommended CD4-guided repeat LTBI screening. Fourth, many patients did not undergo annual IGRA screening for LTBI, although some patients had multiple IGRA tests performed in a year. Additionally, we did not follow annual CD4 counts of all patients to determine if repeat IGRAs were conducted due to low CD4 counts. Another limitation of this study was the low number of new LTBI events (n = 12), which reduced statistical power for subgroup analyses, limited the detection of modest associations, and led to considerable uncertainty in effect estimates. Furthermore, we lacked data on TB epidemiologic risk factors for all 1898 patients. Finally, our study was conducted in a non-endemic setting for TB, limiting the generalizability of our results.

Future work should assess the effectiveness of annual LTBI IGRA-based screening versus targeted screening based on new TB risk factors and CD4 counts. Moreover, more accurate diagnostic tests for LTBI are needed.

## CONCLUSION

In 2 diverse clinics in non-TB endemic settings, the incidence of developing LTBI in PWH was rare among those with negative baseline IGRA. Our findings, showing few new LTBI cases with universal annual screening, support the targeted approach recommended by current guidelines.

## Supplementary Material

ofag227_Supplementary_Data

## Supplementary Data

Supplementary materials are available at *Open Forum Infectious Diseases* online. Consisting of data provided by the authors to benefit the reader, the posted materials are not copyedited and are the sole responsibility of the authors, so questions or comments should be addressed to the corresponding author.
