# Supplementary material for: Is There Value in Performing Yearly Screening for Latent Tuberculosis Infection by Interferon-Gamma Release Assay Among Patients Living With HIV in Non-Endemic Settings?
Source: Open Forum Infect Dis. 2026 Jun 19;13(6):ofag227. doi: 10.1093/ofid/ofag227 (PMC13280639; doi:10.1093/ofid/ofag227)
Supplement: ofag227_Supplementary_Data [file ofag227_supplementary_data.docx]

**Supplementary Materials for**

**Is there value in performing yearly screening for latent tuberculosis infection by interferon-gamma release assay among patients living with HIV in non-endemic settings?**

**Supplement Table 1.** Demographics (N=1898)

|  | N | % |
| --- | --- | --- |
| Sex  Female  Male | 649  1249 | 34.2  65.8 |
| Race  American Indian or Alaska Native  Asian  Black or African American  Native Hawaiian/Pacific Islander  White or Caucasian  Other | 12  22  962  3  527  179 | 0.6  1.2  50.7  0.2  27.8  9.4 |
| LatinX ethnicity | 447 | 23.6 |
| Primary language  English  Non-English | 1661  237 | 87.5  12.5 |

Note: some patients reported more than one race.

**Supplement Table 2.** IGRA results and TB predisposing factors

|  | N | % |
| --- | --- | --- |
| 2017 IGRA (n=1161)  Negative  Positive  Indeterminate | 1107  32  22 | 95.3  2.8  1.9 |
| 2018 IGRA (n=1248)  Negative  Positive  Indeterminate | 1218  25  5 | 97.6  2.0  0.4 |
| 2019 IGRA (n=1297)  Negative  Positive  Indeterminate | 1261  32  4 | 97.2  2.5  0.3 |
| 2020 IGRA (n=1061)  Negative  Positive  Indeterminate | 1013  35  13 | 95.5  3.3  1.2 |
| 2021 IGRA (n=1263)  Negative  Positive  Indeterminate | 979  37  8 | 95.6  3.6  0.8 |
| Any positive IGRA | 112 | 5.9 |
| TB predisposing factors  Diabetes  Smoking use  Substance use  Hepatitis C  Alcohol abuse | 450  330  329  294  174 | 23.7  17.4  17.3  15.5  9.2 |

Note: Yearly IGRA results reflect the number of tests falling into each result category within a given calendar year. The designation "any positive IGRA" refers to the number of unique individuals who had at least one positive IGRA result during the study period. Notably, patients with positive IGRAs in more than one year may be counted in the yearly totals for each relevant year.

**Supplement Table 3.** Features of patients with conversion to a positive or indeterminate QuantiFERON

|  | Negative to positive (N=39) | Indeterminate to positive (N=1) | Negative to indeterminate (N=32) |
| --- | --- | --- | --- |
| Age at seroconversion (mean ± SD) | 53.3 ± 12.7 | 57 | 49.5 ± 14.4 |
| Sex  Female  Male | 12 (30.8%)  27 (69.2%) | 1 (100.0%)  0 (0.0%) | 11 (30.4%)  21 (65.6%) |
| Race/ethnicity  Black  Other  White | 19 (48.7%)  10 (25.6%)  10 (25.6%) | 1 (100.0%)  0 (0.0%)  0 (0.0%) | 19 (59.4%)  4 (12.5%)  9 (28.1%) |
| LatinX ethnicity | 9 (23.1%) | 0 (0.0%) | 4 (12.5%) |
| Charlson CI (mean ± SD) | 2.3 ± 2.2 | 2.0 | 3.8 ± 3.6 |
| CD4 before seroconversion  <200 cells/mm^3^  200-500 cells/mm^3^  >500 cells/mm^3^ | 1 (2.6%)  32 (82.1%)  6 (15.4%) | 0 (0.0%)  1 (100.0%)  0 (0.0%) | 10 (31.2%)  15 (46.9%)  7 (21.9%) |
| CD4 after seroconversion  <200 cells/mm^3^  200-500 cells/mm^3^  >500 cells/mm^3^ | 2 (5.1%)  29 (74.4%)  8 (20.5%) | 0 (0.0%)  0 (0.0%)  1 (100.0%) | 11 (34.4%)  14 (43.8%)  7 (21.9%) |
| History of AIDS | 5 (12.8%) | 0 (0.0%) | 14 (43.8%) |
| HIV viral load (mean ± SD)  Before seroconversion  After seroconversion | 2336 ± 9121  483 ± 2789 | UD  UD | 101893 ± 276324  94098 ± 294454 |
| On ART  Before seroconversion  After seroconversion | 35 (89.7)  38 (97.4) | 1 (100.0%)  1 (100.0%) | 26 (81.2%)  27 (84.4%) |
| Chest radiography  Completed  Granuloma or cavity | 31 (79.5%)  1 (3.2%) | 0 (0.0%)  - | 20 (62.5%)  2 (10.0%) |
| CT chest  Completed  Granuloma | 8 (20.5%)  2 (25.0%) | 0 (0.0%)  - | 14 (43.8%)  3 (21.4%) |
| TB epidemiologic risk factors  Born endemic  Travel endemic  Incarceration  Homelessness  TB exposure  Military service  Healthcare worker  IVDU | 6 (15.4%)  5 (12.8%)  9 (23.1%)  8 (20.5%)  1 (2.6%)  2 (5.1%)  1 (2.6%)  12 (30.8%) | 0 (0%)  0 (0%)  0 (0%)  0 (0%)  0 (0%)  0 (0%)  1 (100.0%)  0 (0%) | 1 (3.1%)  6 (18.8%)  9 (28.1%)  8 (25.0%)  0 (0.0%)  3 (9.4%)  2 (6.2%)  12 (37.5%) |
| TB skin testing  Completed  Positive result | 23 (59.0%)  11 (47.8%) | 1 (100.0%)  0 (0%) | 14 (43.8%)  1 (7.1%) |
| Repeat IGRA result  Negative  Positive  Indeterminate  Not repeated | 17 (43.6%)  10 (25.6%)  0 (0.0%)  12 (30.8%) | 0 (0%)  1 (100.0%)  0 (0%)  0 (0%) | 22 (68.8%)  0 (0.0%)  2 (6.2%)  8 (25.0%) |
| Past treated LTBI | 8 (20.5%) | 0 (0%) | 1 (3.1%) |
| New LTBI diagnosis | 12 (30.8%) | 0 (0%) | 0 (0%) |
| LTBI treatment  Recommended  Started  Completed | 13 (33.3%)  12 (30.8%)  10 (25.6%) | 0 (0%)  -  - | 1 (3.1%)  1 (3.1%)  1 (3.1%) |
| Treatment regimen  INH  INH/RPT | 11 (87.5%)  1 (12.5%) | - | 1 (100.0%)  0 (0.0%) |

CT: computed tomography.

UD: undetectable.

**Supplement Table 4.** Comparing patients negative to positive vs negative to indeterminate seroconversions

|  | Negative to positive (N=39) | Negative to indeterminate (N=32) | p-value |
| --- | --- | --- | --- |
| Charlson CI (mean ± SD) | 2.3 ± 2.2 | 3.8 ± 3.6 | 0.09 |
| CD4 before seroconversion  <200  200-500  >500 | 1 (2.6%)  6 (15.4%)  32 (82.1%) | 10 (31.2%)  7 (21.9%)  15 (46.9%) | 0.002  0.55  0.003 |
| CD4 after seroconversion  <200  200-500  >500 | 2 (5.1%)  8 (20.5%)  29 (74.4%) | 11 (34.4%)  7 (21.9%)  14 (43.8%) | 0.002  1.00  0.01 |
| History of AIDS | 5 (12.8%) | 14 (43.8%) | 0.006 |
| VL (mean ± SD)  Before seroconversion  After seroconversion | 2336 ± 9121  483 ± 2789 | 101893 ± 276324  94098 ± 294454 | 0.002  0.004 |
| On ART  Before seroconversion  After seroconversion | 35 (89.7%)  38 (97.4%) | 26 (81.2%)  27 (84.4%) | 0.33  0.08 |
| Chest radiography  Completed  Granuloma or cavity | 31 (79.5%)  1 (3.2%) | 20 (62.5%)  2 (10.0%) | 0.18  - |
| CT chest  Completed  Granuloma | 8 (20.5%)  2 (25.0%) | 14 (43.8%)  3 (21.4%) | 0.04  - |
| TB risk and predisposing factors  Born endemic country  Travel endemic country  Incarceration  Homelessness  TB exposure  Smoking use  Heavy alcohol use  Diabetes  Malnutrition  Military service  Healthcare worker  Substance use history | 6 (15.4%)  5 (12.8%)  9 (23.1%)  8 (20.5%)  1 (2.6%)  25 (64.1%)  10 (25.6%)  8 (20.5%)  4 (10.3%)  2 (5.1%)  1 (2.6%)  12 (30.8%) | 1 (3.1%)  6 (18.8%)  9 (28.1%)  8 (25.0%)  0 (0%)  25 (78.1%)  7 (21.9%)  12 (37.5%)  8 (25.0%)  3 (9.4%)  2 (6.2%)  12 (37.5%) | 0.20  0.53  0.79  0.78  1.00  0.30  0.79  0.18  0.12  0.65  0.59  0.62 |
| TB skin testing  Done  Positive | 23 (59.0%)  11 (47.8%) | 14 (43.8%)  1 (7.1%) | 0.24  0.02 |
| Repeat IGRA  Negative  Positive  Indeterminate  Not repeated | 17 (43.6%)  10 (25.6%)  0 (0.0%)  12 (30.8%) | 22 (68.8%)  0 (0%)  2 (6.2%)  8 (25.0%) | 0.06  0.002  0.20  0.79 |
| Past treated LTBI | 8 (20.5%) | 1 (3.1%) | 0.04 |
